# Phytochemical composition and cultivar‐dependent variation in colored lettuce (*Lactuca sativa*) and their *α*‐amylase inhibitory activity

**DOI:** 10.1002/jsfa.70994

**Published:** 2026-08-26

**Authors:** Neil Patrick Uy, Hak‐Dong Lee, Sang‐Yun Lee, Hoon Kim, Sanghyun Lee

**Affiliations:** ^1^ Department of Plant Science and Technology Chung‐Ang University Anseong Republic of Korea; ^2^ Department of Food and Nutrition Anyang University Anyang Republic of Korea; ^3^ Natural Product Institute of Science and Technology Anseong Republic of Korea

**Keywords:** phytochemical composition, cultivar variation, LC–MS/MS, HPLC quantification, lettuce

## Abstract

**BACKGROUND:**

This study investigated cultivar‐dependent variation in the phytochemical composition of lettuce (*Lactuca sativa*) using liquid chromatography–tandem mass spectrometry (LC–MS/MS) profiling and high‐performance liquid chromatography (HPLC) quantification, with a focus on enzyme inhibitory activity.

**RESULTS:**

Significant differences were observed among six colored cultivars, with red cultivars exhibiting higher levels of phenolic compounds, particularly miquelianin (**2**), chicoric acid (**4**), and quercetin 3‐*O*‐malonylglucoside (**3**). Quantitative analysis revealed that these metabolites were markedly enriched in red‐pigmented cultivars. Multivariate analysis further confirmed a clear compositional separation between red and green cultivars. *α*‐Amylase inhibitory activity was also evaluated, and the red leaf crude extract yielded the highest efficacy among the matrices (IC_50_ = 1.24 mg mL⁻¹). Among the pure constituents, quercetin 3‐*O*‐malonylglucoside (**3**) demonstrated the strongest enzymatic inhibition (IC_50_ = 18.57 μg mL⁻¹). Molecular docking analysis supported these findings, revealing that compound **3** possessed the highest binding affinity (–9.8 kcal mol⁻¹) toward the *α*‐amylase catalytic site by interacting with key active residues.

**CONCLUSION:**

These findings demonstrate that phytochemical composition and enzyme inhibitory activity in *L. sativa* are strongly influenced by cultivar type. Particularly, red‐pigmented cultivars represent promising sources of bioactive compounds with potential applications in nutritional and functional food development. © 2026 The Author(s). *Journal of the Science of Food and Agriculture* published by John Wiley & Sons Ltd on behalf of Society of Chemical Industry.

## INTRODUCTION

Lettuce (*Lactuca sativa*) is a widely consumed leafy vegetable worldwide, valued for its culinary versatility and its nutritional and functional properties.^1^ Widely recognized as a source of fiber, vitamins, and minerals, lettuce has recently attracted attention for its diverse array of secondary metabolites, particularly polyphenols and flavonoids.^2^ The phytochemical composition of plants can vary considerably depending on genetic, environmental, and developmental factors.^3^ Among cultivated vegetables, *L. sativa* exhibits substantial varietal diversity in leaf color, texture, and biochemical composition.^4^ Characterizing this variation is essential to identify cultivars with enhanced functional potential.

Polyphenols and flavonoids play key roles in plant defense and contribute to human health. These compounds are especially relevant in the context of metabolic disorders, as they can modulate oxidative stress and inhibit carbohydrate‐hydrolyzing enzymes such as *α*‐glucosidase and *α*‐amylase.^5^ These enzymes play a central role in postprandial blood glucose regulation, and their inhibition is a well‐established therapeutic strategy for managing type 2 diabetes.^6^ The dietary intake of natural enzyme inhibitors therefore represents a promising complementary approach to glycemic control. Although previous studies have investigated the *α*‐amylase inhibitory capacity of *L. sativa*, these investigations have primarily evaluated green cultivars.

As far as the authors are aware, no prior research has systematically contrasted various cultivars to connect their pigmentation‐dependent phytochemical profiles directly with their respective amylase inhibitory activities. This study addresses this gap by providing a comprehensive, comparative evaluation across diverse colored *L. sativa* cultivars, distinguishing it from previous studies that have primarily focused on standard green matrices or carotenoids such as lactucaxanthin.^7^ This work also integrates liquid chromatography–tandem mass spectrometry (LC–MS/MS) and high‐performance liquid chromatography–ultraviolet (HPLC‐UV) analyses to characterize the phenolic composition of different color variants of *L. sativa*, while simultaneously assessing the *α*‐amylase inhibitory potential both of crude extracts and their major individual compounds through *in vitro* and *in silico* approaches.

In this study, colored lettuce cultivars were investigated using a multidisciplinary framework. Total phenolic content (TPC) and total flavonoid content (TFC) were determined, and bioactivity was evaluated using an *in vitro* enzyme inhibition assay. Phytochemical profiling was conducted using LC–MS/MS, and key metabolites were quantified by HPLC. Identified compounds were further analyzed through molecular docking to predict their binding affinity to *α*‐amylase. This was followed by *in vitro* validation of their inhibitory effects.

## MATERIALS AND METHODS

### Plant materials and cultivation conditions

Six powdered lettuce (*L. sativa*) cultivars (Fig. 1), namely Red Leaf, Green Leaf, Red Romaine, Romaine, Red Butterhead, and Green Oakleaf, were obtained in May 2023 from the Department of Food Sciences, National Institute of Crop and Food Science, Rural Development Administration, Wanju, Republic of Korea (35.8425 °N, 127.0436 °E). They were authenticated by Dr H. Kim from the same institute.

**Figure 1:**
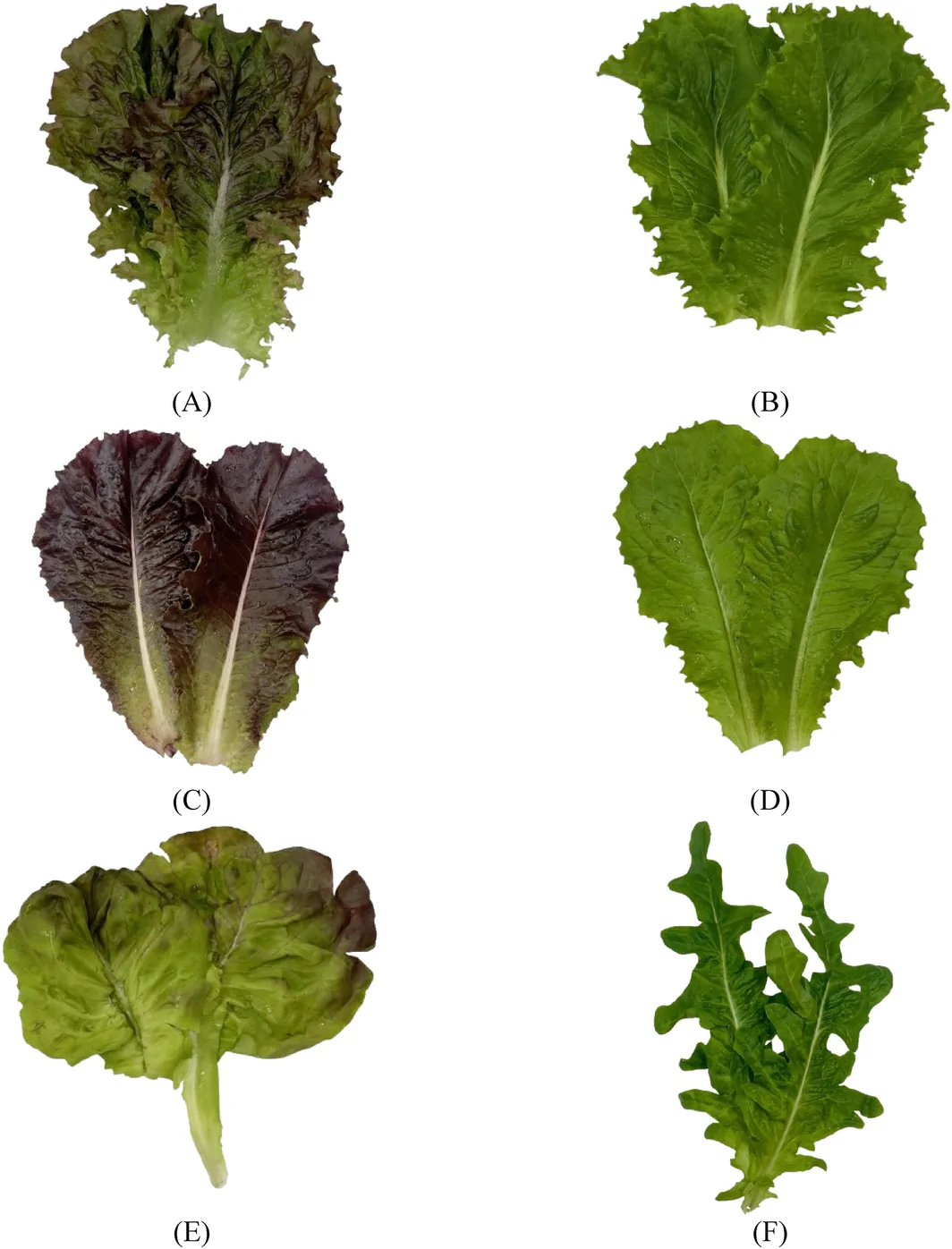
Representative *Lactuca sativa* cultivars among the six studied: (A) Red Leaf, (B) Green Leaf, (C) Red Romaine, (D) Romaine, (E) Red Butterhead, and (F) Green Oakleaf.

All cultivars were cultivated following the institute's standard practices. Seeds were sown in commercial nursery trays containing horticultural substrate and subsequently transplanted into single‐row greenhouse beds covered with black polyethylene mulch film to conserve soil moisture and suppress weed growth. Plants were spaced approximately 20 cm apart within each row, with a distance of approximately 1 m between adjacent beds. During the cultivation period, greenhouse temperatures were maintained at 22–25 °C during the day and 17–20 °C at night under natural photoperiod conditions, with relative humidity ranging from 50% to 75%. Irrigation and fertilization were uniformly supplied to all cultivars through an automated drip irrigation system according to the standard nutrient management practices of the Rural Development Administration.^8^ Environmental conditions, including water and nutrient supply, were controlled carefully throughout the experiment to minimize variation among cultivars. Plants were cultivated in soil under conventional agronomic management practices, including regular irrigation, fertilization, and pest control. Fully expanded leaves were harvested at the commercial maturity stage in May 2023 and transported immediately to the laboratory for subsequent analyses.

### Chemicals and reagents

Folin–Ciocâlteu reagent, sodium carbonate (Na_2_CO_3_), aluminum chloride hexahydrate (AlCl_3_·6H_2_O), and *α*‐amylase from porcine pancreas were purchased from Sigma‐Aldrich Korea (Seoul, Republic of Korea). High‐performance liquid chromatography‐grade solvents, including methanol (MeOH) and water, were purchased from Honeywell Burdick & Jackson (Charlotte, NC, USA). Phosphoric acid and formic acid were obtained from Thermo Fisher Scientific (Waltham, MA, USA), and acetonitrile (ACN) from Scharlau (Sentmenat, Barcelona, Spain). The extraction solvent, 95% ethanol (EtOH), was supplied by Samchun Pure Chemical Co., Ltd. (Pyeongtaek, Republic of Korea). Standards of lactucin (**1**), miquelianin (**2**), quercetin 3‐*O*‐malonylglucoside (**3**), chicoric acid (**4**), and lactucopicrin (**5**) were provided by the Natural Product Institute of Science and Technology (www.nist.re.kr), Anseong, Republic of Korea (Fig. S1).

### Sample extraction methods

For extract preparation, 1 g of each *L. sativa* sample was mixed with 10 mL of absolute EtOH and subjected to ultrasonic extraction at 60 °C for 3 h. The resulting solutions were then filtered, concentrated using a rotary evaporator under reduced pressure to remove solvent and stored until further analysis.

### Total phenolic content assay

Total phenolic content was determined using a previously reported method.^8^ Stock solutions of each extract were prepared and serially diluted to obtain three to five concentrations. For each dilution, 60 μL was added to a well of a 96‐well microplate, followed by 40 μL of Folin–Ciocâlteu reagent and 100 μL of 7.5% Na_2_CO_3_ solution. The plate was incubated at 37 °C for 30 min in the dark, after which absorbance was measured at 760 nm using a microplate reader (Epoch) (BioTek, Winooski, VT, USA). Total phenolic content values were calculated from a standard curve generated using tannic acid (*y* = 0.008*x* − 0.018).

### Total flavonoid content assay

Total flavonoid content was determined using a slightly modified version of a previously described method.^9^ Each extract was diluted from stock solutions to obtain three to five concentrations. Then, 100 μL of each dilution was added to a 96‐well microplate, followed by 100 μL of 2% aluminum chloride hexahydrate solution. The mixture was allowed to react for 10 min at room temperature, after which absorbance was measured at 430 nm using the same microplate reader. Total flavonoid content values were calculated from a standard curve prepared using quercetin solutions (*y* = 0.008*x* − 0.018).

### *In vitro α*‐amylase inhibition assay

The *α*‐amylase inhibitory activity of the extracts was evaluated using a modified colorimetric method.^10^ In a 96‐well microplate, 20 μL of 0.02 mol dm^−3^ phosphate buffer was combined with 20 μL of each test sample or acarbose (positive control) and pre‐incubated with 20 μL of *α*‐amylase (2 U mL^−1^) solution at 50 °C for 15 min. A starch solution was then added as the substrate, and the mixture was incubated for 30 min. The reaction was terminated by adding 96 mmol dm^−3^ 3,5‐dinitrosalicylic acid reagent, followed by heating in a boiling water bath for 15 min to develop color. After cooling, absorbance was measured at 540 nm using a microplate reader. A blank containing buffer instead of the sample was used as the control. The percentage inhibition was calculated using the following equation:

$$\text{inhibition}\;\left(\%\right)=\left(\text{blank}\;{\mathrm{OD}}_{540}–\text{sample}\;{\mathrm{OD}}_{540}\right)/\text{blank}\;{\mathrm{OD}}_{540}\times 100$$

### Determination of the half‐maximal inhibitory concentration (IC_50_ ) and inhibition type

The IC_50_ of each inhibitor was determined using nonlinear regression with a four‐parameter logistic (4PL) model.^11^ The relationship between inhibitor concentration and percentage inhibition was fitted to the following equation:

$$Y=D+\left(A-D\right)/{\left[(1+\left(X/I{\text{C}}_{50}\right)\right]\;}^{B}$$

where *Y* is the response, *X* is the inhibitor concentration, *A* and *D* represent the minimum and maximum asymptotes, respectively, and B is the Hill slope coefficient. Curve fitting was performed using GraphPad Prism version 10.2.3 (GraphPad Software, Boston, MA, USA) by inputting individual data points from all three independent biological replicates (*n* = 3) simultaneously to maximize model rigor. The goodness‐of‐fit for each sigmoid dose–response curve was evaluated and reported using the coefficient of determination (*R* ^2^). The type of inhibition was evaluated using Lineweaver–Burk plots.^12^ Inhibitors were tested at concentrations of 0, 100, 200, and 400 μg mL⁻¹ together with substrate concentrations of 0.5, 1, 2, 4, and 8 mg mL⁻¹. Linear regression of 1/*V* versus 1/[S] was used to determine *K*_*m*_ and *V*_*max*_ values at each inhibitor concentration. The inhibition constant (*K*_*i*_) was subsequently calculated from changes in *K*_*m*_ or *V*_*max*_, according to the inhibition model indicated by the data.

### Liquid chromatography–tandem mass spectrometry sample preparation and conditions

Prior to the analysis, 50 mg of the sample was dissolved in 1 mL of MeOH. Liquid chromatography was performed using a Thermo Vanquish ultra‐high‐performance liquid chromatography (UHPLC) system equipped with a Waters Cortecs T3 column (150 × 2.1 mm, 1.6 μm, Waters Corporation, Milford, MA, USA). The column temperature was maintained at 45 °C. Separation was achieved using a binary solvent system consisting of 0.1% formic acid in water (eluent A) and ACN containing 0.1% formic acid (eluent B), delivered at a flow rate of 0.25 mL min⁻¹ under gradient conditions. Detection was carried out using a Triple TOF 5600+ mass spectrometer (Applied Biosystems SCIEX, Framingham, MA, USA) equipped with a heated electrospray ionization source operating in both positive and negative ion modes. Full‐scan data were acquired over an *m/z* range of 100 to 1500 using a quadrupole mass analyzer at a resolution of 70 000. In positive ion mode, the spray voltage was set to 3.5 kV. The 15 most abundant precursor ions were selected for MS/MS fragmentation at a resolution of 17 500. Additional source parameters included a capillary temperature of 320 °C, sheath gas at 50 AU, auxiliary gas at 10 AU, and sweep gas at 0 AU.

### Preparation of samples and standard solutions for high‐performance liquid chromatography–ultraviolet analysis

Lettuce extracts, along with lactucin (**1**), miquelianin (**2**), quercetin 3‐*O*‐malonylglucoside (**3**), chicoric acid (**4**), and lactucopicrin (**5**), were dissolved in MeOH to prepare stock solutions at concentrations of 30 mg mL⁻¹ for the extracts and 1 mg mL⁻¹ for the standards. The solutions were sonicated for 20 min and filtered through polyvinylidene fluoride (PVDF) membrane filters with a pore size of 0.45 μm. The standard solutions were further diluted to appropriate concentrations for quantitative analysis.

### High‐performance liquid chromatography–ultraviolet analysis conditions

High‐performance liquid chromatography–ultraviolet (HPLC‐UV) analysis was performed using an Agilent 1260 Infinity II Quaternary system equipped with a pump, autosampler, and variable wavelength detector (Santa Clara, CA, USA). Quantitative analysis of the lettuce samples was carried out on a reverse‐phase system fitted with a YMC Pack‐Pro C18 column (250 mm × 4.6 mm, 5 μm particle size), maintained at 30 °C. The mobile phase consisted of 0.1% phosphoric acid in water (solvent A) and 0.1% phosphoric acid in ACN (solvent B). The gradient elution program was as follows: 88% A from 0 to 5 min, 80% A at 15 min, 67% A at 20 min, 50% A at 28 min, 0% A from 31 to 36 min, and 12% B from 38 to 52 min. The flow rate was set at 1.0 mL min⁻¹, and the injection volume was 10 μL. Detection was performed at 260 nm.

### Calibration curve

Standard solutions of lactucin (**1**), miquelianin (**2**), quercetin 3‐*O*‐malonylglucoside (**3**), chicoric acid (**4**), and lactucopicrin (**5**) were serially diluted to prepare at least five concentrations for generating calibration curves. Linearity was assessed using the coefficient of determination (*R* ^2^), and the concentration of each compound in the extracts was calculated using the corresponding calibration equation. In each model, the *x*‐axis represented concentration (μg mL⁻¹), whereas the *y*‐axis corresponded to peak area. Reported values are expressed as means ± standard deviations (SD, *n* = 3). The limits of detection (LOD) and quantification (LOQ) were calculated based on the linear regression model and response factor per unit concentration. These values may be higher than those obtained using conventional signal‐to‐noise approaches (e.g., signal‐to‐noise ratios of 3.3 or 10), as this method reflects variability associated with the overall quantification process rather than instrument sensitivity alone.

### Molecular docking

The phytochemicals quantified in *L. sativa* cultivars were evaluated for their potential interactions with the target protein *α*‐amylase using AutoDock Vina 1.2.0.^13^ The three‐dimensional structures of the ligands were generated by converting their simplified molecular input line entry system (SMILES) notations obtained from the PubChem database (https://pubchem.ncbi.nlm.nih.gov/) into Protein Data Bank (PDB) format using SwissTargetPrediction (https://www.swisstargetprediction.ch/). The protein structures were retrieved from the Research Collaboratory for Structural Bioinformatics (RCSB) Protein Data Bank (https://www.rcsb.org/) in PDB format and prepared by removing water molecules and other nonessential components using PyMOL.^14^ Docking simulations were performed using the prepared protein and ligand structures. The resulting ligand–protein interactions were visualized using Biovia Discovery Studio Visualizer (https://www.3ds.com/products/biovia/discovery-studio).^15^

### Statistical analysis

GraphPad Prism version 8.0.2 was used for statistical analyses and data visualization. Data were obtained from three independent experiments, each conducted in triplicate, and are presented as means ± SDs. Before analysis, datasets were tested for normality and log‐normal distribution. One‐way analysis of variance (ANOVA) was used to assess differences among groups, followed by Tukey's multiple comparison test for *post hoc* analysis. A significance level of *P* < 0.05 was applied. Lineweaver–Burk plots were generated using Microsoft Office Excel 2019. Correlation analysis and principal component analysis (PCA) were conducted to evaluate relationships among phenolic composition, TPC, TFC, and *α*‐amylase inhibitory activity in red and green *L. sativa* cultivars. Heatmap visualization and clustering were performed using MetaboAnalyst (version 6.0).^16^

## RESULTS

### Total phenolic content and TFC assays

The TPC and TFC of the colored *L. sativa* cultivars were determined to evaluate variations across the samples (Fig. 2). The results revealed clear diversity among them. The Red Leaf cultivar exhibited the highest TPC at 47.62 mg TAE g⁻¹, significantly exceeding that of the other groups, whereas the Green Oakleaf showed the lowest TPC at 21.35 mg TAE g⁻¹ extract, indicating a comparatively lower phenolic content.

**Figure 2:**
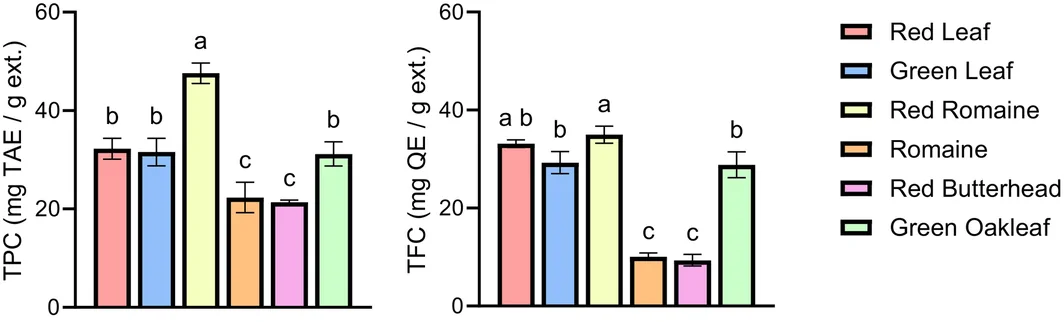
Total phenolic content (TPC, mg TAE g⁻¹ extract) and total flavonoid content (TFC, mg QE g⁻¹ extract) of six *Lactuca sativa* cultivars. Values are presented as mean ± SD (*n* = 3). Different lower case letters indicate significant differences.

The TFC results followed a similar trend. Red Leaf showed the highest flavonoid content at 34.96 mg quercetin equivalents QE g⁻¹ extract, followed by Red Butterhead (33.13 mg QE/g extract) and Red Romaine (29.26 mg QE/g extract). In contrast, Romaine and Green Oakleaf exhibited the lowest flavonoid contents, with values of 10.08 and 9.33 mg QE g⁻¹ extract, respectively.

### *In vitro α*‐amylase inhibition of *L. sativa* extracts

The antidiabetic potential of the colored *L. sativa* extracts and target compounds was evaluated using an *in vitro α*‐amylase inhibition assay. In general, inhibitory activity increased in a dose‐dependent manner with extract concentration (Fig. 3). Among the samples tested, Red Leaf, Red Romaine, and Red Butterhead showed the strongest *α*‐amylase inhibitory effects. Green Leaf and Romaine exhibited moderate activity, whereas Green Oakleaf showed the weakest inhibition. Acarbose, used as the positive control, exhibited substantially stronger inhibition, reflecting its established efficacy as an *α*‐amylase inhibitor.

**Figure 3:**
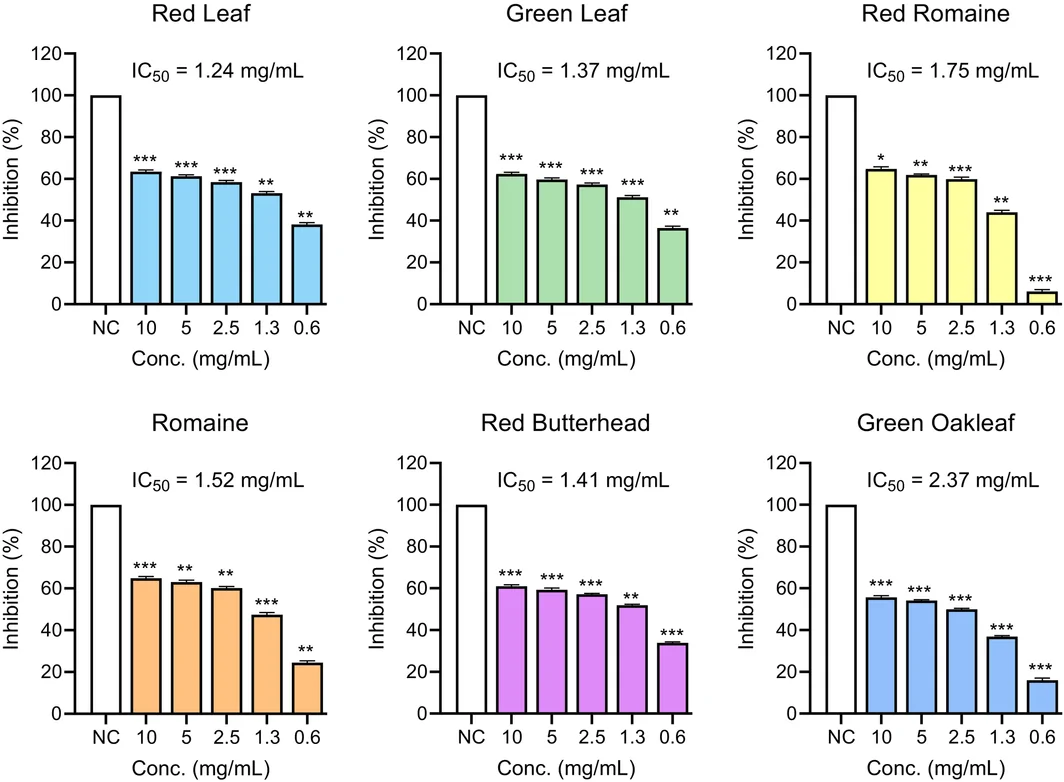
Inhibitory effects of *Lactuca sativa* extracts on *α*‐amylase activity. Activity is expressed as percentages relative to the control. Data are presented as means ± SDs of three independent experiments. ***P < 0.001, **P < 0.01, and *P < 0.05 in comparison with the control.

As observed in the dose–response profiles, the maximum inhibitory activity of the crude extracts plateaued at approximately 60% to 70% even at the highest tested concentration (10 mg mL⁻¹). This sub‐maximal ceiling reflects the complex, unpurified nature of the raw plant extracts, where noninhibitory matrix components dilute the target specialized metabolites. For quantitative comparison, the IC_50_ values for the crude extracts are detailed directly in the panels of Fig. 3. The Red Leaf extract demonstrated the highest potency among the raw matrices with an IC_50_ value of 1.24 mg mL⁻¹, followed by Green Leaf (1.37 mg mL⁻¹), Red Butterhead (1.41 mg mL⁻¹), Romaine (1.52 mg mL⁻¹), and Red Romaine (1.75 mg mL⁻¹). Conversely, Green Oakleaf displayed the weakest profile with an IC_50_ value of 2.37 mg mL⁻¹. In comparison, the target compounds and acarbose yielded significantly lower IC_50_ values.

### Phytochemical profiling by LC–MS/MS analysis

Liquid chromatography–mass spectrometry analysis in negative ionization mode provided a comprehensive overview of the phytochemical composition of the samples, revealing a structurally diverse range of secondary metabolites (Supporting Information, Table S1). Compound identification was based on accurate mass measurements, calculated molecular formulas, and characteristic retention times, supported by diagnostic MS/MS fragmentation patterns (Supporting Information, Fig. S2). Most compounds generated stable deprotonated molecular ions [M − H]^−^, which underwent characteristic fragmentation reactions, including neutral losses of sugar moieties such as glucose (−162 Da) and glucuronic acid (−176 Da), ester‐linked groups such as caffeoyl (−162 Da), and acyl substituents such as acetyl (−42 Da) and malonyl (−86 Da). These fragmentation pathways produced structurally informative ions, often corresponding to the aglycone or phenolic core of the molecule. Base peaks in the MS/MS spectra, representing the most abundant product ions, typically corresponded to these aglycones or quinic acid derivatives and served as key indicators for tentative compound identification. Interpretation of these fragmentation patterns, supported by literature reports and phytochemical databases, enabled confident structural assignment even in the absence of authentic reference standards.

Five phenolic acids were identified with high confidence. Caftaric acid exhibited fragmentation consistent with the loss of tartaric acid, producing a base peak at *m/z* 179 corresponding to deprotonated caffeic acid. Similarly, caffeoylquinic acid generated fragment ions at *m/z* 191 (quinic acid) and *m/z* 179 (caffeic acid), supporting its identification as a chlorogenic acid isomer. Caffeic acid itself showed a prominent base peak at *m/z* 179 corresponding to its deprotonated molecular ion. Chicoric acid exhibited sequential neutral losses of two caffeic acid moieties, yielding fragment ions at *m/z* 311 and 179, consistent with reported fragmentation patterns.

Flavonoid glycosides such as spiraeoside and miquelianin showed base peaks at *m/z* 301, corresponding to the quercetin aglycone formed after cleavage of glucose and glucuronic acid moieties, respectively. These compounds also produced secondary ions at *m/z* 179 and 151, characteristic of retro‐Diels–Alder fragmentation of the flavonoid backbone. Scutellarin and luteolin 4′‐glucoside generated fragment ions at *m/z* 285, representing the luteolin aglycone. Quercetin derivatives, including quercetin 3‐*O*‐malonylglucoside and quercetin 3‐(6″‐acetylglucoside), produced ions at *m/z* 301 following sequential losses of sugar and acyl groups. 1,3‐Dicaffeoylquinic acid showed fragmentation with ions at *m/z* 353 and 191, confirming the presence of two caffeoyl groups and a quinic acid core. Other phenolic compounds included esculin, which produced fragment ions at *m/z* 179 and 161, characteristic of coumarin glucosides. In the later stages of the chromatogram, the sesquiterpene lactones lactucopicrin and lactucin exhibited more complex fragmentation patterns but retained characteristic ions associated with their guaianolide skeletons, including *m/z* 247 and 229, attributed to the loss of water and small functional groups from the lactone ring. Finally, genistin showed a typical loss of glucose, yielding a base peak at *m/z* 269, consistent with its structure as a glycosylated isoflavone.

### Quantitative analysis of compounds 1–5 by HPLC‐UV

High‐performance liquid chromatography–ultraviolet (HPLC‐UV) analysis of the six *L. sativa* cultivars achieved good separation of the standard compounds, with well‐defined retention times (Supporting Information, Table S2). Although LC–MS/MS profiling identified a total of 15 constituents, the quantitative HPLC analysis focused on compounds **1**–**5** whose identities were authentically confirmed by retention time and spectral matching with commercially available reference standards. The remaining ten constituents detected by LC–MS/MS were either below the LOQ under HPLC‐UV conditions or lacked commercial reference standards for absolute quantification. The results revealed significant variation in the concentration and distribution of key secondary metabolites, namely lactucin (**1**), miquelianin (**2**), quercetin 3‐*O*‐malonylglucoside (**3**), chicoric acid (**4**), and lactucopicrin (**5**) (Table 1). Compounds were identified based on ultraviolet (UV) absorbance characteristics and retention time matching with reference standards, and quantification was performed using calibration curves constructed from known concentrations (Supporting Information, Fig. S3).

**Table 1:** Content of compounds **1**–**5** in different *Lactuca sativa* cultivars

Sample	Content (mg/g DW)					
	**1**	**2**	**3**	**4**	**5**	Total
Red Leaf	ND	0.823 ± 0.003	4.187 ± 0.009	2.359 ± 0.009	0.028 ± 0.001	7.40
Green Leaf	tr	0.392 ± 0.002	1.103 ± 0.004	1.022 ± 0.003	0.024 ± 0.001	2.54
Red Romaine	tr	0.370 ± 0.008	3.083 ± 0.014	1.555 ± 0.002	0.066 ± 0.001	5.07
Romaine	0.011 ± 0.012	0.295 ± 0.009	0.316 ± 0.003	0.434 ± 0.001	0.021 ± 0.001	1.08
Red Butterhead	ND	0.606 ± 0.015	2.852 ± 0.013	1.682 ± 0.002	0.021 ± 0.001	5.16
Green Oakleaf	0.043 ± 0.002	tr	0.146 ± 0.001	0.387 ± 0.003	0.143 ± 0.001	0.72

ND, not detected; tr, trace. Compounds: lactucin (**1**), miquelianin (**2**), quercetin 3‐*O*‐malonylglucoside (**3**), chicoric acid (**4**), and lactucopicrin (**5**).

Red Leaf exhibited the highest total content of these compounds, primarily due to elevated levels of quercetin 3‐*O*‐malonylglucoside (**3**) and chicoric acid (**4**), along with moderate levels of miquelianin (**2**) and trace amounts of lactucopicrin (**5**); lactucin (**1**) was not detected. Red Butterhead also showed high levels of miquelianin (**2**), quercetin 3‐*O*‐malonylglucoside (**3**), and chicoric acid (**4**), with low lactucopicrin (**5**) and no detectable lactucin (**1**). Red Romaine ranked slightly lower but maintained appreciable levels of all compounds except lactucin (**1**), which was present only in trace amounts. In contrast, Green Oakleaf showed the lowest overall metabolite content, with minimal levels of all detected compounds and no detectable miquelianin (**2**). Romaine and Green Leaf exhibited intermediate to low total content, with modest levels of quercetin 3‐*O*‐malonylglucoside (**3**) and chicoric acid (**4**), variable amounts of miquelianin (**2**), and trace levels of lactucin (**1**) and lactucopicrin (**5**).

### Hierarchical clustering heatmap

Hierarchical clustering of the cultivars based on their phytochemical profiles, TPC, TFC, and *α*‐amylase inhibitory activity revealed a clear separation between red and green *L. sativa* cultivars (Fig. 4). Red cultivars clustered together and were characterized by higher relative levels of chicoric acid (**4**), quercetin 3‐*O*‐malonylglucoside (**3**), and miquelianin (**2**), whereas green cultivars formed a separate cluster with comparatively lower levels of these compounds. In contrast, lactucin (**1**) and lactucopicrin (**5**) contributed less to the differentiation between groups. This clustering pattern suggests that flavonoid‐rich profiles, particularly the accumulation of quercetin derivatives and chicoric acid (**4**), are the primary drivers of cultivar differentiation and are likely associated with the enhanced *α*‐amylase inhibitory activity observed in red cultivars.

**Figure 4:**
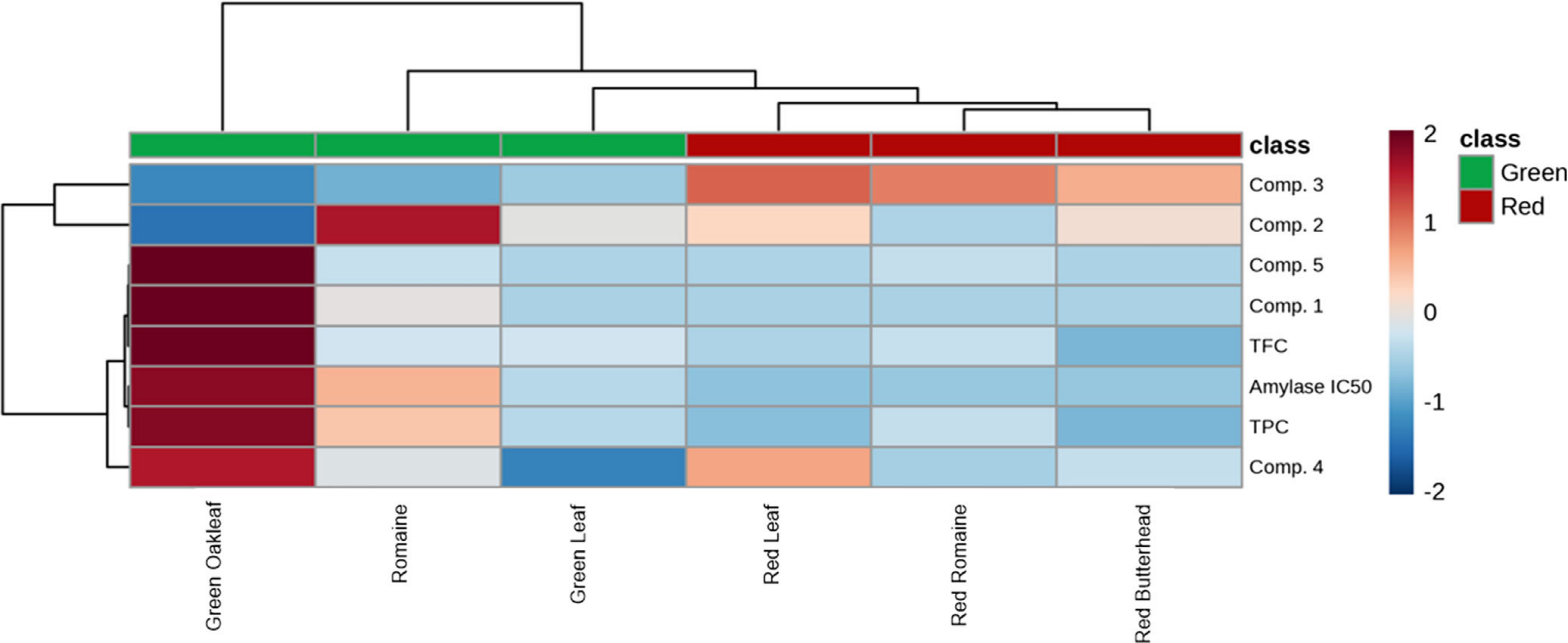
Correlation heatmap with hierarchical clustering of phytochemical contents in the six *Lactuca sativa* cultivars. The clustering pattern highlights associations between polyphenolic profiles and biological activity. Compounds: lactucin (**1**), miquelianin (**2**), quercetin 3‐*O*‐malonylglucoside (**3**), chicoric acid (**4**), and lactucopicrin (**5**).

### Principal component analysis

Principal component analysis confirmed the separation between red and green cultivars observed in the hierarchical clustering (Fig. 5). The first principal component (PC1), which explained 95.6% of the total variance, clearly separated red cultivars on the positive side from green cultivars on the negative side. The second principal component (PC2) accounted for 4.2% of the variance and contributed only minor additional separation within each group. The tight clustering of red cultivars indicates high compositional similarity, whereas green cultivars showed greater variability, largely driven by differences in TPC and TFC values. Loadings from PC1 indicate that variables such as TPC, TFC, and flavonoid glycosides contribute most strongly to the observed separation, suggesting that these compounds are key determinants of both cultivar classification and enzyme inhibitory potential.

**Figure 5:**
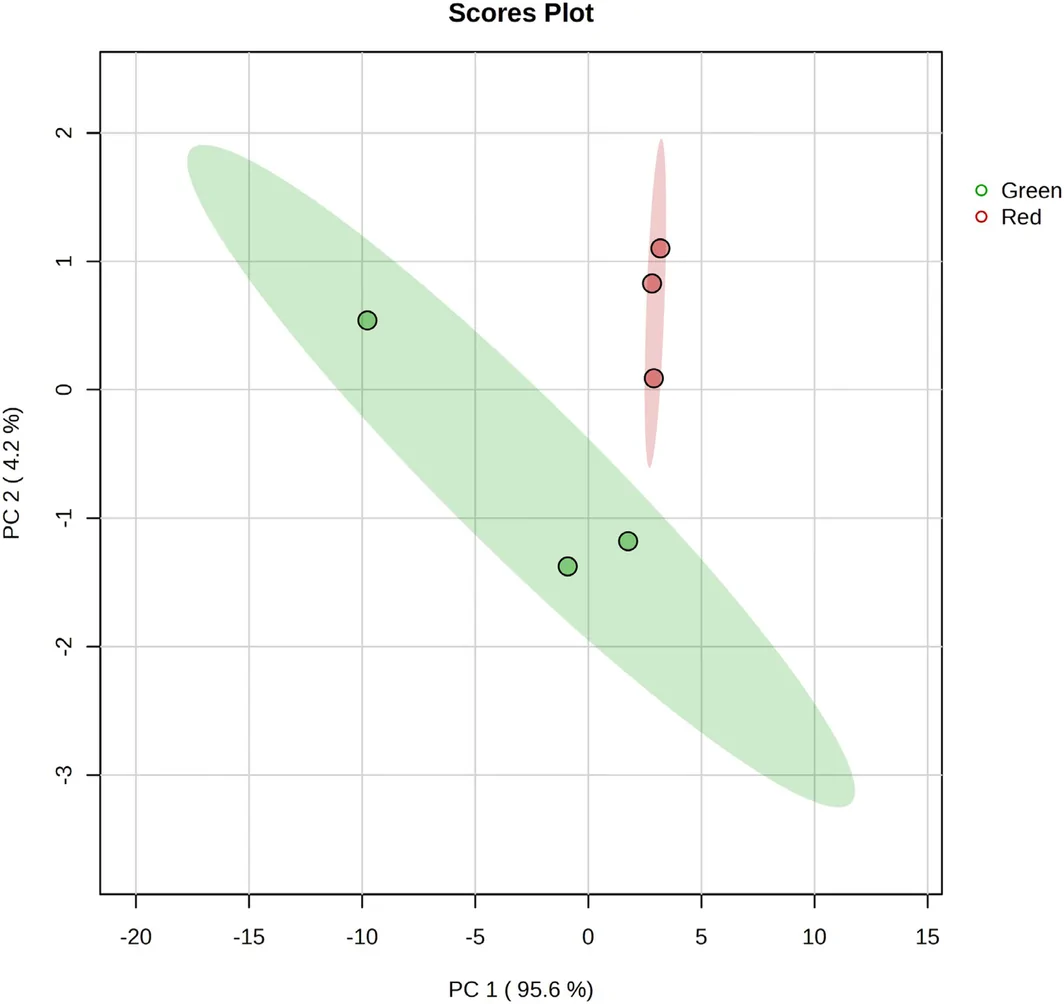
Principal component analysis (PCA) biplot of six *Lactuca sativa* cultivars based on phytochemical composition and *α*‐amylase inhibitory activity. Red and green cultivars form distinct clusters, reflecting differences in phenolic content and associated bioactivity.

### *In vitro α*‐amylase inhibition of compounds 1–5

The *α*‐amylase inhibitory activity of the target compounds was evaluated to assess their potential as natural antidiabetic agents (Fig. 6). All compounds inhibited *α*‐amylase activity in a concentration‐dependent manner over the range of 9.37 to 300 μg mL⁻¹. The IC_50_ values indicated substantial variation in inhibitory potency. Quercetin 3‐*O*‐malonylglucoside (**3**) exhibited the strongest inhibition, with an IC_50_ of 18.57 μg mL⁻¹, followed by miquelianin (**2**) and lactucopicrin (**5**), with IC_50_ values of 25.71 μg mL⁻¹ and 32.37 μg mL⁻¹, respectively. Chicoric acid (**4**) showed moderate activity (IC_50_ = 37.03 μg mL⁻¹), while lactucin (**1**) was the least potent, with an IC_50_ of 42.18 μg mL⁻¹. These values were higher than that of the positive control, acarbose (IC_50_ = 2.29 μg mL⁻¹), indicating comparatively lower inhibitory potency. Lineweaver–Burk plots revealed distinct inhibition patterns among the tested compounds (Supporting Information, Fig. S4). Lactucin (**1**) exhibited noncompetitive inhibition, characterized by a relatively constant *K*_*m*_ with a change in *V*_*max*_. Quercetin 3‐*O*‐malonylglucoside (**3**) and lactucopicrin (**5**) exhibited competitive inhibition, as indicated by an increase in the apparent *K*_*m*_ with little to no change in *V*_*max*_, producing lines that intersect at the y‐axis. In contrast, miquelianin (**2**) and chicoric acid (**4**) showed mixed‐type inhibition, characterized by simultaneous changes in both *K*_*m*_ and *V*_*max*_, resulting in lines that intersect away from the axes. These classifications were based on differences in slopes and intercepts across inhibitor concentrations.

**Figure 6:**
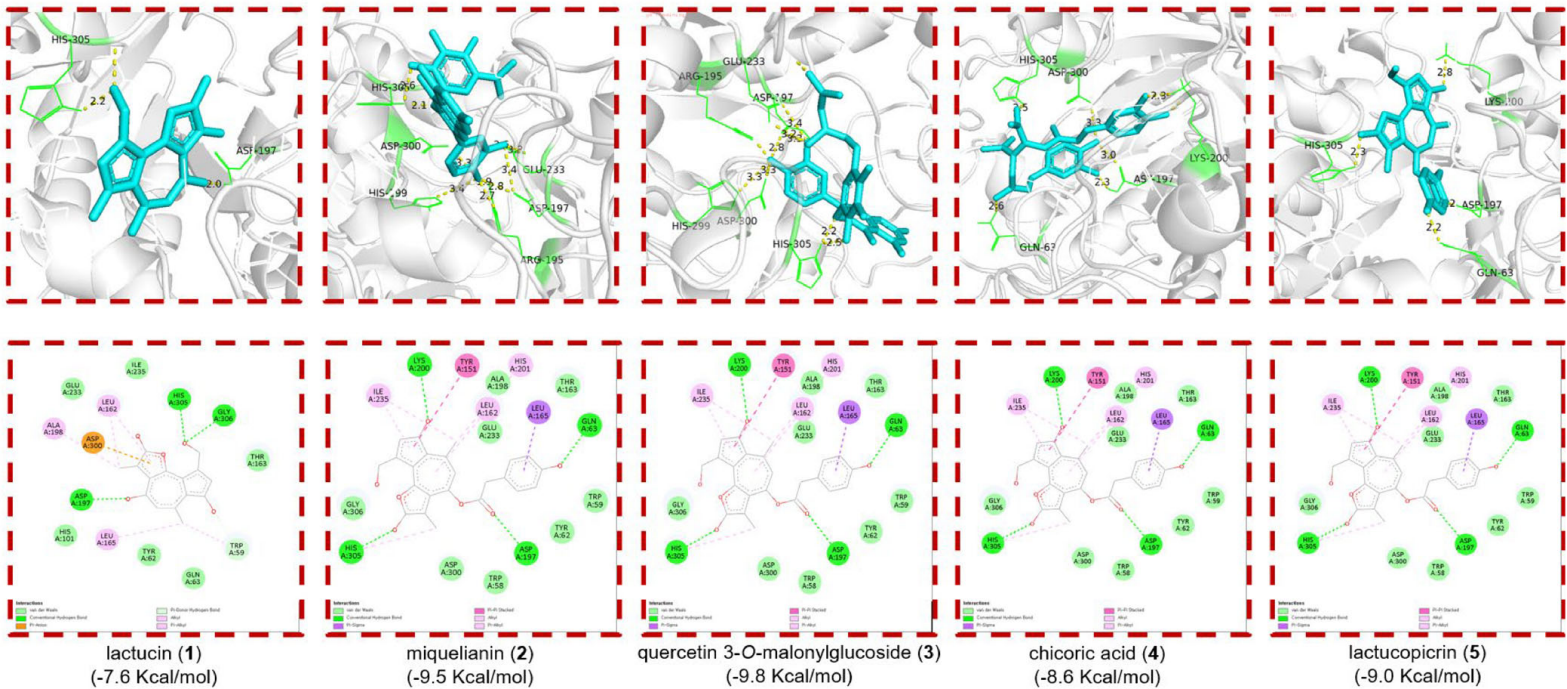
Three‐dimensional interactions of compounds **1–5** with *α*‐amylase. The binding affinities of each ligand to the enzyme are shown. Compounds: lactucin (**1**), miquelianin (**2**), quercetin 3‐*O*‐malonylglucoside (**3**), chicoric acid (**4**), and lactucopicrin (**5**).

### Molecular docking simulations

Docking simulations were performed for the five compounds – lactucin (**1**), miquelianin (**2**), quercetin 3‐*O*‐malonylglucoside (**3**), chicoric acid (**4**), and lactucopicrin (**5**) – to evaluate their binding affinity and interactions with the *α*‐amylase active site (Fig. 7). Binding affinities ranged from −7.6 to −9.8 kcal mol⁻¹. Quercetin 3‐*O*‐malonylglucoside (**3**) showed the strongest binding affinity at −9.8 kcal mol⁻¹ and formed multiple hydrogen bonds with key catalytic residues, including Asp197, Glu233, and His305. This was followed by miquelianin (**2**) and lactucopicrin (**5**), with binding affinities of −9.5 and − 9.0 kcal mol⁻¹, respectively. Miquelianin (**2**) interacted with residues Asp197, Glu233, and Tyr62, whereas lactucopicrin (**5**) also interacted with residues within the catalytic site. Chicoric acid (**4**) exhibited a binding affinity of −8.6 kcal mol⁻¹ and interacted with Glu233, Asp300, and His305. Lactucin (**1**) exhibited the weakest binding affinity at −7.6 kcal mol⁻¹, forming fewer interactions with the catalytic site and lacking strong hydrogen bonding interactions.

**Figure 7:**
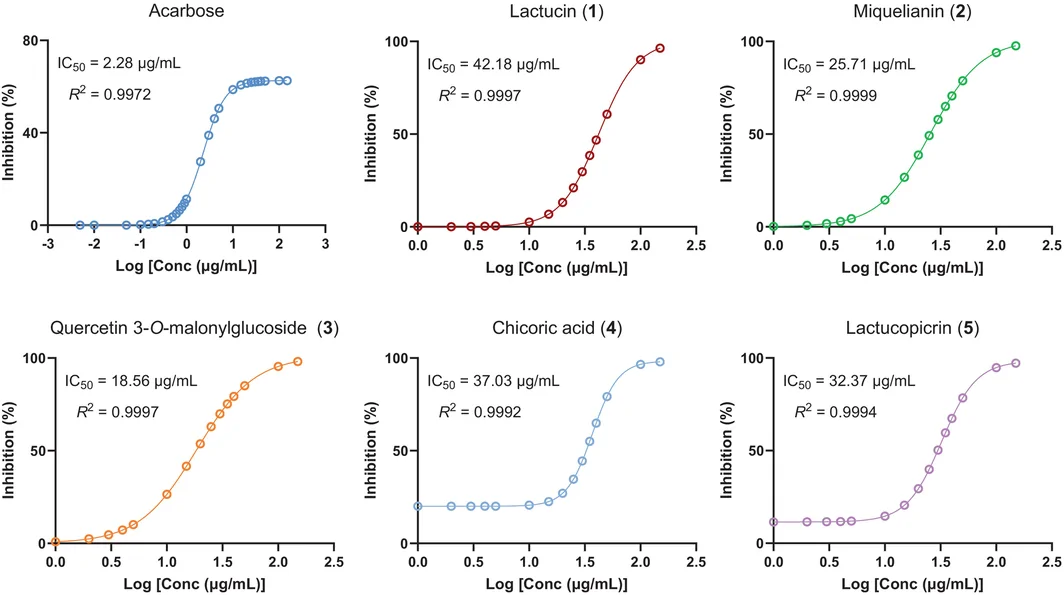
Dose–response curves and IC_50_ values of acarbose and compounds **1–5** on *α*‐amylase activity. Inhibition was plotted against the log‐transformed inhibitor concentrations and fitted using the nonlinear regression 4PL model.

## DISCUSSION

This study evaluated the antidiabetic potential of colored *L. sativa* cultivars by integrating phytochemical profiling, *α*‐amylase inhibition assays, and molecular docking simulations. The results demonstrate that the biological activity of *L. sativa* is influenced by substantial biochemical variation among cultivars, even within a single edible species that is often regarded primarily as a dietary vegetable. Although the cultivars examined belong to the same botanical species, their *α*‐amylase inhibitory activity differed considerably, indicating that cultivar selection can influence biological function beyond conventional nutritional parameters.^17^

The results of the TPC and TFC analyses suggest that secondary metabolism contributes directly to the observed variation in enzyme inhibition. This observation is consistent with previous studies showing that lettuce cultivars with higher phenolic content generally exhibit stronger biological activity. Gan and Azrina reported that Red Coral lettuce contained the highest TPC among five cultivars, significantly exceeding Green Coral, Romaine, Butterhead, and Iceberg.^18^ Similarly, Shi *et al*. found that the red cultivar, Lollo Rosso exhibited higher TPC and TFC values than green cultivars.^19^ These findings indicate that visible differences among cultivars are often associated with broader metabolic variation, as also observed in other phenolic‐rich vegetables.^20^ The stronger inhibitory activity observed in phenolic‐rich cultivars suggests that these metabolites may contribute to the suppression of carbohydrate digestion.^21^ This relationship is consistent with previous reports in which phenolic‐rich lettuce extracts exhibited significant *α*‐amylase inhibitory activity.^22^ In contrast, an earlier study proposed that carotenoid constituents such as lactucaxanthin are the primary contributors to the antidiabetic activity of *L. sativa*. The present results indicate that polyphenolic compounds may play a more prominent role in the inhibitory effects observed among these cultivars.^23^ Total phenolic measurements can therefore serve as useful preliminary indicators, whereas detailed metabolite profiling provides a more accurate basis for interpreting biological activity.

The variation in inhibitory activity among cultivars also suggests that enzyme inhibition depends not only on TPC but also on the presence and relative abundance of specific bioactive compounds. A previous study about *L. sativa* populations has shown that bioactivity often fails to correlate directly with TPC, but instead tracks closely with specific individual metabolites, most notably caffeoylquinic acid isoforms and specific flavonoid conjugates such as quercetin‐3‐malonylglucoside‐7‐glucuronide and kaempferol‐3‐malonylglucoside.^24^ Although chicoric acid and quercetin‐3‐malonylglucoside typically dominate the lettuce phenolic pool, subtle variations in minor or conjugated metabolites frequently drive observed differences in biological activity across genotypes due to transgressive segregation. Untargeted LC–MS/MS profiling was conducted to further examine this relationship, and it showed that all cultivars shared a broadly similar phytochemical framework while differing in the relative abundance of individual constituents. The detection of hydroxycinnamic acids, flavonoid glycosides, coumarin derivatives, and sesquiterpene lactones confirms that *L. sativa* contains a chemically diverse metabolite profile. The predominance of caffeoyl derivatives and flavonoid conjugates is consistent with previous metabolomic studies of lettuce, in which phenylpropanoid‐derived compounds were identified as major constituents. Chicoric acid (**4**) has been consistently reported as one of the principal phenolic acids, particularly in red cultivars.^25^ Similarly, quercetin 3‐*O*‐malonylglucoside (**3**) has been identified as a characteristic flavonoid in pigmented lettuce cultivars.^26^ The frequent detection of fragment ions corresponding to caffeic acid and quercetin‐related structures suggests that these compounds form a central phytochemical backbone across the cultivars. Rather than showing that all detected metabolites contribute equally, the LC–MS/MS profile indicates that specific phenolic subclasses are more closely associated with the observed inhibitory activity. The concurrent presence of sesquiterpene lactones alongside phenolic compounds further suggests that multiple biosynthetic pathways may contribute to the functional properties of lettuce.^25^

The quantitative HPLC analysis provided stronger evidence that differences in biological activity are associated with variations in the concentration of specific compounds rather than with total metabolite abundance alone. Cultivars exhibiting stronger *α*‐amylase inhibition contained higher levels of miquelianin (**2**) and quercetin 3‐*O*‐malonylglucoside (**3**), indicating that these metabolites likely contribute substantially to the observed activity. This observation is consistent with previous studies showing that elevated concentrations of flavonoid glycosides are often associated with enhanced antioxidant and antidiabetic properties in leafy vegetables.^27^ Furthermore, the relatively low abundance of lactucin (**1**) and lactucopicrin (**5**) should not be interpreted as evidence of limited biological relevance, as compounds present at low concentrations can still exert significant effects when they possess high intrinsic potency or act synergistically with more abundant metabolites.^28^ This distinction is important, as it indicates that metabolite concentration alone does not determine functional importance.

Multivariate analyses consistently distinguished red and green *L. sativa* cultivars, highlighting strong associations between phytochemical composition and cultivar type. Red cultivars were enriched in chicoric acid (**4**) and flavonoid glycosides such as quercetin 3‐*O*‐malonylglucoside (**3**) and miquelianin (**2**), which aligns with the stronger *α*‐amylase inhibitory activity observed in biochemical assays. These findings are consistent with previous reports linking anthocyanin‐ and flavonoid‐rich red lettuce cultivars to enhanced antidiabetic properties.^29^ The tight clustering of red cultivars suggests a relatively uniform phytochemical profile dominated by bioactive flavonoids. The same metabolites that contribute to separation in PCA are also associated with stronger enzyme inhibition, indicating that the multivariate patterns reflect biologically meaningful variation rather than purely statistical differences. In contrast, green cultivars exhibited greater heterogeneity, with variation largely driven by TPC and TFC values, reflecting lower flavonoid enrichment. Their broader distribution in PCA space indicates variability in phenolic composition, which may underlie differences in bioactivity. Overall, the separation between red and green cultivars underscores the predictive value of phytochemical signatures for distinguishing lettuce cultivars and supports the use of metabolomics‐based approaches to link cultivar type with functional properties.

To further elucidate the mechanism underlying *α*‐amylase inhibition, molecular docking simulations were performed. The docked compounds exhibited varying degrees of interaction with the enzyme's catalytic site, primarily through hydrogen bonding and aromatic interactions with residues such as Asp197, Glu233, and His305. Quercetin 3‐*O*‐malonylglucoside (**3**) and miquelianin (**2**) demonstrated strong binding affinities and formed stable interactions within the active site, suggesting high compatibility with the enzyme pocket.^30^ These compounds formed multiple hydrogen bonds with the target protein, indicating favorable binding orientation and stability.^31^ This interaction pattern is consistent with their strong *in vitro* inhibitory activity and supports a direct relationship between molecular binding and functional enzyme inhibition. In contrast, lactucin (**1**) exhibited weaker binding and fewer stabilizing interactions, likely due to the absence of polar or aromatic features required for strong enzyme–ligand interactions. These structural differences help explain the variation in inhibitory potency among the compounds.

The activity of the target compounds provides further evidence that molecular structure strongly influences enzyme inhibition. Quercetin 3‐*O*‐malonylglucoside (**3**) showed the strongest inhibitory activity and the most favorable docking affinity, suggesting that its structural features are well suited for interaction with the *α*‐amylase catalytic region. The flavonol scaffold contains multiple hydroxyl groups capable of forming hydrogen bonds with residues involved in substrate recognition, and the malonyl group may enhance polar interactions within the active site.^32^ Although direct reports describing the *α*‐amylase inhibitory activity of these specific flavonoid glycosides remain limited, their close structural similarity to quercetin, a well‐established *α*‐amylase inhibitor, suggests that much of their activity arises from the conserved quercetin aglycone backbone.^33^ This interpretation is consistent with previous findings showing that flavonoids bearing multiple hydroxyl substitutions often exhibit stronger inhibition of digestive enzymes. In contrast, the weaker inhibition and lower docking affinity observed for lactucin (**1**) differ from some earlier reports in which sesquiterpene lactones showed notable enzyme inhibitory activity, indicating that not all members of a phytochemical class contribute equally.^34^ These results support a structure–activity relationship in which subtle variations in molecular architecture can lead to meaningful differences in biological performance.

The influence of glycosylation on inhibitory potency is also noteworthy. Glycosylated flavonoids, such as miquelianin (**2**) and quercetin 3‐*O*‐malonylglucoside (**3**), retained substantial inhibitory activity despite the presence of sugar residues that are sometimes considered to reduce direct interaction with enzymes. This observation is consistent with studies suggesting that glycosylation can enhance water solubility and improve the accessibility of polyphenols to enzyme surfaces in aqueous environments.^35^ Conversely, other studies have reported reduced activity following glycosylation, as sugar substitution can mask reactive hydroxyl groups on the flavonoid nucleus.^36^ The present findings suggest that the effect of glycosylation depends more on the position and nature of the sugar moiety than on glycosylation itself. In particular, the strong activity of quercetin 3‐*O*‐malonylglucoside (**3**) indicates that acylated glycosides may achieve a favorable balance between solubility and binding affinity. This provides a more nuanced perspective than the common assumption that glycosylated flavonoids are inherently less active than their aglycone forms.

The enzyme kinetic analysis indicates that the inhibitory effects of lettuce metabolites are mechanistically diverse. The presence of competitive, noncompetitive, and mixed‐type inhibition among the compounds suggests that the extract can interfere with *α*‐amylase through multiple molecular pathways. Competitive inhibition by quercetin 3‐*O*‐malonylglucoside (**3**) suggests direct interaction with the catalytic site, whereas mixed‐type inhibition by miquelianin (**2**) and chicoric acid (**4**) reflects interactions with both the free enzyme and the enzyme–substrate complex. Such mechanistic diversity may enhance the overall functional effect of the crude extract, as inhibition can occur under a range of substrate conditions. Previous studies have often attributed plant‐derived *α*‐amylase inhibition to a single dominant metabolite,^37^ whereas the present findings suggest that the activity of *L. sativa* is more likely driven by cooperative interactions among multiple compounds. This supports the possibility of synergistic inhibition, in which compounds with distinct modes of action collectively produce stronger effects than would be expected from their individual contributions alone.

Taken together, these results indicate that the antidiabetic potential of *L. sativa* depends on a complex interplay between metabolite abundance, molecular structure, and collective phytochemical behavior. Biological activity is therefore not determined solely by higher phenolic content, but also by the enrichment of specific metabolites with favorable structural features and complementary inhibitory mechanisms. In particular, the strong inhibitory activity and binding affinity observed for quercetin 3‐*O*‐malonylglucoside (**3**) highlight this flavonoid glycoside as a key contributor to the *α*‐amylase inhibitory properties of lettuce extracts. The present study provides new evidence linking this compound to the antidiabetic potential of *L. sativa*, extending previous work that has primarily focused on broader phenolic classes rather than individual metabolites. The consistency among phytochemical profiling, quantitative analysis, enzyme kinetics, molecular docking, and chemometric modeling offers a coherent explanation for the observed cultivar‐dependent differences. These results suggest that Red Romaine and Red Leaf cultivars may represent promising candidates for functional food applications aimed at glycemic control. Nevertheless, further *in vivo* studies are required to determine whether the inhibitory effects observed under experimental conditions translate into meaningful physiological benefits following dietary intake.

Collectively, the findings of this study indicate that cultivar selection plays a critical role in shaping both phytochemical composition and associated functional properties in lettuce. The combined LC–MS/MS profiling and HPLC quantification provide a robust compositional dataset that can serve as a reference for future studies on lettuce quality and nutritional evaluation.

## CONCLUSIONS

This study demonstrated substantial variability in phytochemical composition and biological activity among colored *L. sativa* cultivars. Red‐pigmented cultivars exhibited significantly higher TPC and TFC levels, which were associated with stronger *α*‐amylase inhibitory activity and higher binding affinity in molecular docking simulations. Among the identified compounds, quercetin 3‐*O*‐malonylglucoside (**3**), miquelianin (**2**), and lactucopicrin (**5**) showed the highest bioactivity, indicating their potential as functional components for glycemic control, pending further validation. The integration of LC–MS/MS profiling, *in vitro* enzyme assays, and molecular docking provides a robust framework for the identification and evaluation of natural antidiabetic agents from dietary sources. These results highlight the relevance of specific colored *L. sativa* cultivars as sources of bioactive phytochemicals associated with enzyme inhibition. However, as this study is limited to *in vitro* and *in silico* analyses, the physiological relevance of these results remains to be established. Future research should focus on *in vivo* validation, as well as on the bioavailability, stability, metabolism, and digestibility of key metabolites, to better assess their potential role in glycemic control.

## CONFLICT OF INTEREST

The authors declare no conflict of interest.

## AUTHOR CONTRIBUTIONS

Neil Patrick Uy: investigation, writing – original draft preparation. Hak‐Dong Lee: investigation, data curation. Sang‐Yun Lee: investigation, data curation. Hoon Kim: methodology, data curation. Sanghyun Lee: conceptualization, supervision, writing – review and editing.

## Supporting information


**Table S1.** Tentative identification of compounds in *L. sativa* by LC–MS/MS analysis in negative ionization mode.
**Table S2.** Calibration data for lactucin (**1**), miquelianin (**2**), quercetin 3‐*O*‐malonylglucoside (**3**), chicoric acid (**4**), and lactucopicrin (**5**).
**Figure S1.** Chemical structures of lactucin (**1**), miquelianin (**2**), quercetin 3‐*O*‐malonylglucoside (**3**), chicoric acid (**4**), and lactucopicrin (**5**).
**Figure S2.** The UV (a) and base peak (b) chromatogram of *L. sativa* sample (negative mode).
**Figure S3.** HPLC‐UV chromatograms of compounds **1**–**5** (A) and EtOH extracts of Red Leaf (B), Green Leaf (C), and Red Romaine (D). Compounds: lactucin (**1**), miquelianin (**2**), quercetin 3‐*O*‐malonylglucoside (**3**), chicoric acid (**4**), and lactucopicrin (**5**).
**Figure S4.** Lineweaver‐Burk plots illustrating the *α*‐amylase inhibitory activities of lactucin (**1**), miquelianin (**2**), quercetin 3‐*O*‐malonylglucoside (**3**), chicoric acid (**4**), and lactucopicrin (**5**) at concentrations of 0, 100, 200, and 400 μg/mL.

## Data Availability

All data generated or analyzed in this study are included in this article. Additional information is available from the corresponding author upon reasonable request.
