# Supplementary material for: Phytochemical composition and cultivar‐dependent variation in colored lettuce (Lactuca sativa) and their α‐amylase inhibitory activity
Source: J Sci Food Agric. 2026 Aug 26;107(1):422–33. doi: 10.1002/jsfa.70994 (PMC13756578; doi:10.1002/jsfa.70994)
Supplement: Supplementary file 1 [file JSFA-107-422-s001.docx]

**Supplementary Materials**

**Phytochemical composition and cultivar-dependent variation in colored lettuce (*Lactuca sativa*) and their α-amylase inhibitory activity**

Neil Patrick Uy, Hak-Dong Lee, Sang-Yun Lee, Hoon Kim, Sanghyun Lee

**Table S1**

Tentative identification of compounds in *L. sativa* by LC-MS/MS analysis in negative ionization mode.

| t_R_ (min) ***^a^*** | Adducts | Precursor *m/z* | Exact mass (Da) | Molecular formula | Tentative identity |
| --- | --- | --- | --- | --- | --- |
| 5.41 | [M-H]^-^ | 311.041 | 312.048 | C_13_H_12_O_9_ | Caftaric acid |
| 7.35 | [M-H]^-^ | 339.073 | 340.079 | C_15_H_16_O_9_ | Esculin |
| 9.04 | [M-H]^-^ | 353.086 | 354.095 | C_16_H_18_O_9_ | Caffeoylquinic acid |
| 9.32 | [M-H]^-^ | 311.041 | 312.048 | C_9_H_8_O_4_ | Caffeic acid |
| 16.01 | [M-H]^-^ | 473.073 | 474.080 | C_22_H_18_O_12_ | Chicoric acid |
| 16.69 | [M-H]^-^ | 463.088 | 464.095 | C_21_H_20_O_12_ | Spiraeoside |
| 17.87 | [M-H]^-^ | 477.067 | 478.075 | C_21_H_18_O_13_ | Miquelianin |
| 18.52 | [M-H]^-^ | 461.073 | 462.080 | C_21_H_18_O_12_ | Scutellarin |
| 18.71 | [M-H]^-^ | 447.093 | 448.101 | C_21_H_20_O_11_ | Luteolin 4'-glucoside |
| 19.34 | [M-H]^-^ | 549.089 | 550.096 | C_24_H_22_O_15_ | Quercetin 3-*O*-malonylglucoside |
| 19.36 | [M-H]^-^ | 505.099 | 506.106 | C_23_H_22_O_13_ | Quercetin 3-(6''-acetylglucoside) |
| 19.95 | [M-H]^-^ | 515.119 | 516.127 | C_25_H_24_O_12_ | 1,3-Dicaffeoylquinic acid |
| 20.51 | [M-H]^-^ | 431.098 | 432.106 | C_21_H_20_O_10_ | Genistin |
| 25.15 | [M-H]^-^ | 409.129 | 410.137 | C_23_H_22_O_7_ | Lactucopicrin |
| 25.16 | [M-H-H_2_O]^-^ | 257.062 | 276.100 | C_15_H_16_O_5_ | Lactucin |

**Table S2**

Calibration data for lactucin (**1**), miquelianin (**2**), quercetin 3-*O*-malonylglucoside (**3**), chicoric acid (**4**), and lactucopicrin (**5**)

| Compound | *^a^*t_R_ (min) | Range (mg/mL) | Regression equation | *^b^R*^2^ | *^c^*LOD (µg/mL) | *^d^*LOQ (µg/mL) |
| --- | --- | --- | --- | --- | --- | --- |
| **1** | 15.4 | 0.1–0.00078 | Y = 21.188X **-** 0.0755 | 1.0000 | 0.001 | 0.002 |
| **2** | 22.2 | 0.1–0.00078 | Y = 21.991X **+** 3.3 | 1.0000 | 0.001 | 0.05 |
| **3** | 23.7 | 0.1–0.00078 | Y = 16.939X **+** 7.528 | 0.9997 | 0.001 | 0.002 |
| **4** | 24.6 | 0.1–0.00078 | Y = 11.22X **+** 2.2078 | 0.9999 | 0.001 | 0.002 |
| **5** | 34.3 | 0.1–0.00078 | Y = 15.144X **–** 3.0616 | 1.0000 | 0.002 | 0.005 |

*^a^*retention time; *^b^*coefficient of determination; *^c^*limit of detection; *^d^*limit of quantification

|  |  |  |
| --- | --- | --- |
| **1** | **2** | **3** |
|  | |  |
| **4** | | **5** |

**Figure S1.** Chemical structures of lactucin (**1**), miquelianin (**2**), quercetin 3-*O*-malonylglucoside (**3**), chicoric acid (**4**), and lactucopicrin (**5**).

(a)

(b)

**Figure S2.** The UV (a) and base peak (b) chromatogram of *L. sativa* sample (negative mode)

(A)

(B)

(C)

(D)

**Figure S3.** HPLC-UV chromatograms of compounds **1**–**5** (A) and EtOH extracts of red leaf (B), green leaf (C), and red romaine (D). Compounds: lactucin (**1**), miquelianin (**2**), quercetin 3-*O*-malonylglucoside (**3**), chicoric acid (**4**), and lactucopicrin (**5**).

|  |  |
| --- | --- |
|  |  |
|  | |

**Figure S4.** Lineweaver-Burk plots illustrating the α-amylase inhibitory activities of lactucin (**1**), miquelianin (**2**), quercetin 3-*O*-malonylglucoside (**3**), chicoric acid (**4**), and lactucopicrin (**5**) at concentrations of 0, 100, 200, and 400 μg/mL.
